# Leaves of Yellow Gentian (*Gentiana lutea*) as an Alternative Source of Bitter Secoiridoid Glycosides

**DOI:** 10.1021/acs.jnatprod.2c00529

**Published:** 2022-08-24

**Authors:** Serena Fiorito, Francesco Epifano, Lucia Palumbo, Chiara Collevecchio, Fabrizio Mascioli, Roberto Spogli, Salvatore Genovese

**Affiliations:** # Dipartimento di Farmacia, Università “Gabriele d’Annunzio” of Chieti-Pescara, Via dei Vestini 31, 66100 Chieti Scalo (CH), Italy; § Enrico Toro Distilleria Srl, Via Tiburtina Valeria − Km.142,440, 65028 Tocco da Casauria (PE), Italy; $ Prolabin & Tefarm Srl, Via dell’Acciaio 9, 06134 Perugia, Italy

## Abstract

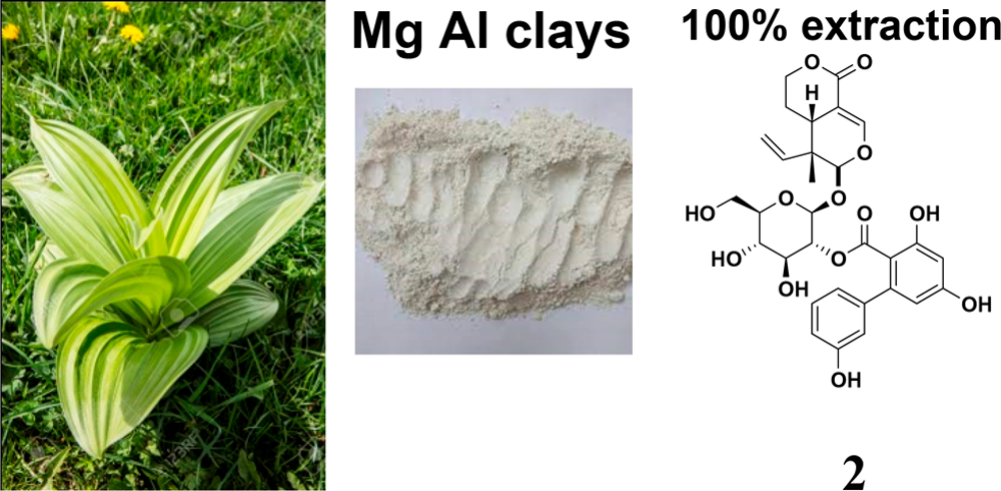

In a search for methods of manufacturing bitter principles from *Gentiana lutea*, mainly represented by gentiopicroside (**1**) and amarogentin (**2**), as an alternative to extraction from the roots of this plant, in this short communication it is shown that the leaves of this plant can be regarded as an additional source of such phytochemicals. Extraction of *G. lutea* leaves was coupled to solid-phase adsorption by differently structured solids as a separation technology step, providing a selective isolation of both these secondary metabolites in good to excellent yields. Thus, the extraction of bitter secoiridoids can be achieved in an equivalent or improved way rather than processing the roots of *G. lutea* while preserving the biodiversity of the species.

Extracts of roots and rhizomes of *Gentiana lutea* L. [Gentianaceae; common name “yellow gentian”; synonyms *Asterias hybrida* G. Don, *Asterias lutea* (L.) Borkh., *Coilantha biloba* Bercht. & J. Presl, *Gentiana major* Bubani, and *Gentianusa lutea* (L.) Pohl] represent a medicinal and healthy remedy used in Western, traditional Chinese, Tibetan, and Ayurvedic medicinal practices and appear in several national and international pharmacopeias as a powerful stomachic agent.^1^ Yellow gentian roots are also the main ingredient of a bitter liqueur widely consumed in Northern and Central Italy and in the Alpine regions of France, Switzerland, Germany, Austria, and Slovenia.^2^ The bitterness of such alcoholic beverages is due mainly to the presence of two secoiridoid glycosides, namely, gentiopicroside (**1**) and amarogentin (**2**) (Figure 1).

**Figure 1:**
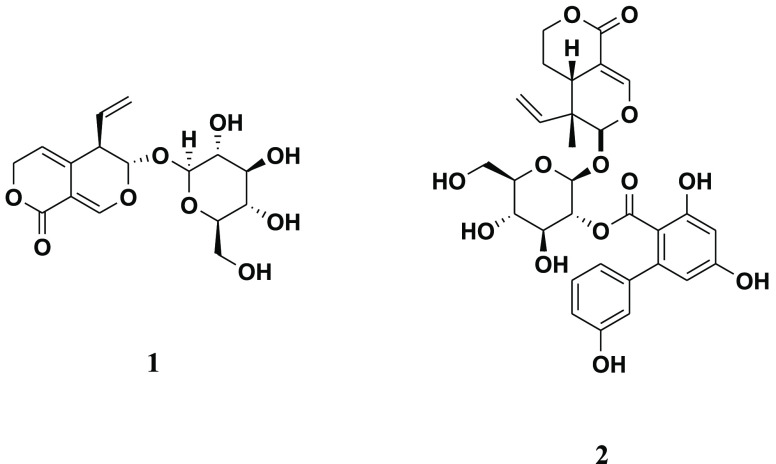
Structures of gentiopicroside (**1**) and amarogentin (**2**).

The values of their respective bitterness indexes, 58 × 10^6^ for amarogentin (**2**) and 12 × 10^3^ for gentiopicroside (**1**), reveal why these two secondary metabolites can be regarded as the most widely used naturally occurring bitter-tasting substances.^3^ Thus, amarogentin (**2**) and gentiopicroside (**1**) are the main determinants of the typical bitter taste of alcoholic beverages obtained traditionally from gentian roots. These are used widely by local populations mostly in the form of homemade liqueurs. Their manufacturing process necessarily involves the collection of large quantities of roots for extraction from plants of at least five years of age. Consequently, there is a substantial risk of a major depletion of these plants in the areas where yellow gentian grows, since the repopulation times in these areas are extremely slow. For these reasons, the collection of yellow gentian roots is strictly regulated by national and regional laws, and the plant is a protected species in several European countries. For example, the collection of yellow gentian is regulated at the EU level under the Council Directive 92/43/EEC of May 21, 1992, on the conservation of natural habitats and of wild fauna and flora, and EU Commission Regulation no. 1320/2014 of December 1, 2014, amending Council Regulation (EC) no. 338/97 on the protection of species of wild fauna and flora by regulating trade. Consequently, enhancements in the technology of the extraction of bitter principles from yellow gentian are desirable to overcome this drawback. The ideal protocol would allow amarogentin (**2**) and gentiopicroside (**1**) to be obtained in good yields, leaving the source plants intact and alive.

To achieve this aim, in this communication the leaves of *G. lutea* were investigated as an alternative source of both bitter secoiridoid glycosides, by evaluating their content with HPLC/DAD methodology in plants originating from mountains of the Abruzzo Region (Central Italy) and collected in the period May–August 2020. Then, their selective extraction from leaf crude ethanolic extracts was investigated by coupling maceration with a solid-phase adsorption step using differently functionalized solids (listed in Table 1), followed by desorption. The main results of the present investigation consisted in having obtained alcoholic blends enriched in gentiopicroside (**1**) and amarogentin (**2**) as potential naturally occurring bitter additives for foods and beverages.

**Table 1:** Solid Sorbents Employed for the Adsorption of Gentiopicroside (**1**) and Amarogentin (**2**) from an Ethanolic Leaf Extract of *G. lutea*

entry^a^	layered double hydroxides
A	Zn Al oleate
B	Zn Al nitrate
C	Zn Al chloride
D	Mg Al nitrate
E	Mg Al azelate
F	Mg Al hydroxide chloride
G	Mg Al hydroxide acetate
H	Mg Al hydroxide carbonate
I	Mg Al acetate
L	Zn hydroxy chloride
	Lamellar Solids
M	Zr(HPO_4_)_2_ type B
N	Zr(HPO_4_)_2_ type B + stearamine
	Oxides/Hydroxides
O	MgO
P	Mg(OH)_2_
	Phyllosilicates
Q	bentonite
R	talc
S	mica L
T	mica F
U	mica SFG20
V	Mg Al benzensulfonate
Z	Zn Al benzenesulfonate

a All solids are commercially available and were provided by Prolabin & Tefarm Srl (Perugia, Italy).

The first set of experiments consisted of the collection, drying, powdering, and extraction with absolute EtOH of *G. lutea* leaves to assess and quantify the presence of amarogentin (**2**) and gentiopicroside (**1**). Some literature communications have suggested that both these secoiridoid glycosides are found in the leaves of some plants belonging to the family Gentianaceae including a subspecies of *G. lutea*, namely, subsp. *symphyandra* Murb.,^4^ and several *Swertia* spp.^5^ Additional information has suggested that these bitter principles are biosynthesized in the leaves and subsequently translocated to and accumulated in the root parenchyma.^6^ Such findings have not been followed up in terms of practical phytotherapeutic uses of yellow gentian leaves.

After an overnight maceration, followed by evaporation of the solvent to complete dryness, and HPLC/DAD analysis, according to the literature,^3^ the recorded concentrations in the leaves of gentiopicroside (**1**) and amarogentin (**2**) were 70.5 ± 0.08 mg/g and 20.6 ± 0.05 mg/g of the dry extract, respectively. Notably, these values are comparable to those obtained from the extraction of roots of the same plant source^3^ for gentiopicroside (**1**) and higher in the case of amarogentin (**2**) (+28.8%). A description of the HPLC analysis procedure, its validation, and a list of the main related analytical parameters are provided in the Supporting Information. Thus, the first set of quantitative data represented a confirmation of already literature reported information about the presence of bitter secoiridoid glycosides in yellow gentian leaves and were supportive of the next step, the solid-phase adsorption experiments with a group of 21 solid sorbents listed in Table 1. Toward this aim, the alcoholic solution extract (21 mL) was divided into 21 aliquots of equal volume, poured into amber vials, and evaporated to dryness under a vacuum. The raw waxy solids so obtained were suspended in double-distilled H_2_O (1 mL) and finally submitted to treatment with the solid-phase material (200 mg) added to each vial. All suspensions were allowed to react overnight at room temperature under magnetic stirring and subsequently filtered. The solids collected on filters were first washed twice with double-distilled H_2_O (5 mL) and finally with absolute EtOH (3 × 5 mL) to accomplish the desorption of gentiopicroside (**1**) and/or amarogentin (**2**) as retained on the sorbents. These filtrates were then analyzed by HPLC/DAD to quantify the bitter principle, and the quantification data are reported in Table 2.

**Table 2:** Quantitative Determination of Gentiopicroside (**1**) and Amarogentin (**2**) (Values Expressed as μg/mL and Percentages ± SD) from Leaf Ethanolic Extracts of *G. lutea* Absorbed onto the Solid Sorbents under Investigation

	**1**		**2**	
entry	μg/mL ± SD	% ± SD	μg/mL ± SD	% ± SD
A	11.1 ± 0.07	33.7 ± 0.3	2.9 ± 0.03	47.5 ± 0.1
B	12.2 ± 0.09	37.1 ± 0.1	5.2 ± 0.04	85.2 ± 0.3
C	11.4 ± 0.07	34.5 ± 0.3	5.6 ± 0.04	91.8 ± 0.1
D	11.9 ± 0.06	36.2 ± 0.1	6.1 ± 0.03	100 ± 0. 2
E	11.4 ± 0.06	34.6 ± 0.4	6.1 ± 0.04	100 ± 0. 1
F	13.9 ± 0.04	42.0 ± 0.1	6.1 ± 0.05	100 ± 0. 2
G	16.5 ± 0.05	50.1 ± 0.2	6.1 ± 0.05	100 ± 0.2
H	11.9 ± 0.07	36.0 ± 0.2	5.8 ± 0.07	95.1 ± 0.2
I	9.2 ± 0.03	27.8 ± 0.2	6.1 ± 0.01	100 ± 0.2
L	13.7 ± 0.08	41.6 ± 0.1	4.4 ± 0.02	72.1 ± 0.1
M	11.2 ± 0.10	33.8 ± 0.2	4.8 ± 0.07	78.7 ± 0.5
N	13.1 ± 0.08	39.7 ± 0.3	4.7 ± 0.06	77.0 ± 0.4
O	12.1 ± 0.14	38.0 ± 0.3	2.8 ± 0.04	45.9 ± 0.2
P	12.5 ± 0.11	38.1 ± 0.1	3.0 ± 0.04	49.2 ± 0.1
Q	18.3 ± 0.09	55.5 ± 0.2	4.5 ± 0.05	73.7 ± 0.1
R	14.1 ± 0.09	42.8 ± 0.2	6.1 ± 0.04	100 ± 0.1
S	29.5 ± 0.15	89.5 ± 0.3	4.7 ± 0.04	77.0 ± 0.3
T	8.5 ± 0.02	25.7 ± 0.1	4.6 ± 0.03	75.4 ± 0.4
U	27.7 ± 0.08	83.9 ± 0.3	1.8 ± 0.01	29.5 ± 0.5
V	6.2 ± 0.03	18.8 ± 0.1	6.1 ± 0.03	100 ± 0.1
Z	7.0 ± 0.03	21.1 ± 0.1	6.2 ± 0.04	100 ± 0.3

An unexpected, peculiar trend for the adsorption of the two secoiridoid glycosides was recorded. In general, all solids exhibited a higher capacity to retain amarogentin (**2**) than gentipicroside (**1**). The percentages of adsorption for compound **1** reached satisfactory values for only two entries out of 21, namely, 89.5% for mica L and 83.9% for mica SFG20, while all other percentages were in the range 18.8–50.1%. In contrast for amarogentin (**2**), excellent results were obtained with nine out of 21 sorbents, which provided percentages of adsorption of 95%, and in most cases quantitative extractive yields were recorded. This was revealed in particular for Mg- and Al-containing solid materials, including Mg Al nitrate (entry D), Mg Al azelate (entry E), Mg Al hydroxy chloride (entry F), Mg Al hydroxy acetate (entry G), Mg Al hydroxy carbonate (entry H), talc (entry R), and Mg Al benzensulfonate (entry V). Zn Al benzensulfonate (entry Z) was the only exception of a clay not containing Mg and displayed similar results to those recorded in this preliminary screening. The greater tendency of Mg- and Al-containing sorbents recorded herein represent a confirmation of already reported data in the literature.^7,8^ More oxyphilic and “harder” Mg and Al metal centers seem to be more prone to interact tightly and coordinate phenolic moieties, as can be found in the structure of both gentian secoiridoids, as opposed to “softer” Zn ones. The differences in structure between gentiopicroside (**1**) and amarogentin (**2**), in the larger number of phenolic hydroxy groups of the latter compound, may account for the considerable differences recorded in adsorption in Table 2.

To confirm the selectivity toward the preconcentration of the two desired secoiridoids from crude yellow gentian leaf extracts, TLC of the desorbed solutions deriving from the treatment with each solid listed in Table 1, using commercially available gentiopicroside (**1**) and amarogentin (**2**) standards as the references, was performed with a mixture of CH_2_Cl_2_–MeOH (7:3) as the mobile phase. After detection with UV (254 nm), I_2_, KMnO_4_, H_2_SO_4_, and phosphomolibdic acid, the presence of the secoiridoids **1** and **2** as the only detected compounds was shown.

As a further step in the investigation, the effect of sorbent loading on extractive yields was considered. Thus, the nine most effective solids resulting from the preliminary screening as described above were selected (entries D–I, R, V, and Z), and increased quantities of the same (from 10 to 100 mg) were employed under identical experimental conditions for extraction and subsequent quantification by HPLC. Amarogentin (**2**) was used as the reference compound, and results are reported in Table 3.

**Table 3:** Effect of Sorbent Loading on Amarogentin (**2**) Adsorption^a^

	sorbent loading			
entry	100 mg	50 mg	25 mg	10 mg
D	100 ± 0.2	100 ± 0.4	100 ± 0.1	94.4 ± 0.2
E	100 ± 0.1	100 ± 0.2	100 ± 0.3	100 ± 0.3
F	100 ± 0.3	100 ± 0.3	100 ± 0.1	83.3 ± 0.4
G	100 ± 0.5	100 ± 0.3	93.0 ± 0.2	76.1 ± 0.1
H	100 ± 0.3	64.4 ± 0.2	44.1 ± 0.2	28.2 ± 0.2
I	100 ± 0.1	100 ± 0.1	98.7 ± 0.4	98.3 ± 0.1
R	100 ± 0.4	62.1 ± 0.3	48.9 ± 0.2	11.7 ± 0.1
V	100 ± 0.2	100 ± 0.3	100 ± 0.1	100 ± 0.1
Z	92.1 ± 0.5	85.4 ± 0.1	83.7 ± 0.2	55.1 ± 0.2

a Percentages of adsorption ± DS.

The data shown in Table 3 indicate clearly how the ability for the total removal of amarogentin (**2**) from extracts of yellow gentian leaves remained practically unaltered for five sorbents (entries D–F, I, and V) out of the nine selected for further investigation. Of these, Mg Al azelate (entry E), Mg Al benzensulfonate (entry V), and Mg Al acetate (entry I) gave quantitative or nearly quantitative adsorption yields with the lowest sorbent loading level (10 mg). All these three solids shared the presence of an organic anion of medium to high lipophilicity intercalated in the lamellar layers.^9^ This seems to greatly facilitate the adsorption and consequently the interaction with organic compounds, like gentiopicroside (**1**) and amarogentin (**2**), presumably due to interactions of a lipophilic nature or of the van der Waals type. The present results confirm a trend exhibited by these same materials (especially by Mg Al azelate, entry E) with other classes of natural products like anthraquinones,^10^ phenolic acids, flavonoids, purine alkaloids,^11^ diarylheptanoids,^12^ capsaicinoids,^13^ oxyprenylated coumarins,^14^ apocarotenoids,^15^ and anthocyanins.^16^

Further changes of experimental parameters and conditions (e.g., a modified operational time and an increase of temperature) led to worse data (e.g., lower extractive yields and chemical degradation) than those described above. Once it was determined that Mg Al azelate (entry E), Mg Al acetate (entry I), and Mg Al benzensulfonate (entry V) were the most effective sorbents, they were each recycled after the first treatment by drying in an oven at 70 °C for 2 h. Five further steps of solid-phase adsorption of amarogentin (**2**) were conducted by adopting the lowest loading (10 mg) and the same experimental conditions as described. The percentages of adsorption obtained were 100%, 100%, 99.8%, 100%, and 99.6% for Mg Al azelate, 100%, 99.9%, 99.9%, 100%, and 99.8% for Mg Al benzensulfonate, and finally 99.2%, 98.7%, 98.8%, 99.1%, and 98.4% for Mg Al acetate. Such values clearly indicate that the solids handled are recyclable and reusable with no loss of their adsorption capacity.

Hence, a preliminary overview of a new extraction technique of secoiridoid glycosides of a high commercial value, like gentiopicroside (**1**) and amarogentin (**2**), is based on the following milestones: (a) use of solid materials featured by easy handling, low cost, easy and high-yielding chemical synthesis, versatile functionalization recyclability, and reusability, (b) good to excellent extractive and preconcentration yields, and (c) use of a renewable plant source. This last aspect of the procedure, as developed herein, is of particular interest considering that gentian is a rare species and subject to environmental protection in practically all the regions where this plant grows. Although, as stated above, few studies have reported the presence of secoiridoids in yellow gentian leaves, the present study, detailing their quantification, has shown that leaves can be regarded as valid and effective sources of bitter principles with respect to roots and finally provides valuable means for their selective preconcentration and extraction in quantitative yields, which does not seem to have been reported in the literature. Thus, the presently described approach aimed at the extraction of bitter secoiridoid glycosides allows generating easily and rapidly purely nature-derived blends with a potential to become basic ingredients for the preparation of gentian-based extracts, but also for pharmaceutical, nutraceutical, and cosmetic purposes. The scheme that has been optimized on a laboratory scale in principle could be transferred to pilot plant and industrial reactor applications. Experiments to assess the effectiveness and capacities of a wider panel of solid materials with different structures and chemicophysical properties are presently ongoing in our laboratories.

## Experimental Section

### General Experimental Procedures

The same general procedure as reported previously was followed for the extraction of plant material, solid-phase adsorption, and HPLC analyses.^3^ Analytical conditions and parameters are detailed in the Supporting Information.

### Plant Material

Leaves of *G. lutea* were collected in Maiella Mountain (Abruzzo region, Italy) in the period May–August 2020 with the permission obtained from local government authorities. Plant samples were properly taxonomically identified by the authors. A voucher specimen (GL-L-2020-1) is stored on the deposit in the laboratory of the Chemistry of Natural Compounds, Department of Pharmacy, University “G. d’Annunzio” of Chieti-Pescara.

### Extraction and Isolation

Leaf extracts were obtained by overnight maceration in absolute EtOH. The experimental protocol consisted of suspending 10 g of finely triturated leaf powder in 120 mL of EtOH followed by filtration and evaporation to complete dryness under a vacuum. The raw waxy solid extract was redissolved in EtOH to reach a final concentration of 1000 ppm. The resulting mixture was divided into 21 aliquots of equal volume (1 mL), followed by evaporation to dryness of the solvent. Each solid so obtained was suspended into H_2_O (5 mL) and treated with quantities of sorbents A–Z indicated in the text above. Each resulting mixture was stirred magnetically overnight at room temperature, filtered, and centrifuged (13000*g*). The solid collected on the filter was washed with absolute EtOH (3 × 5 mL) to accomplish the complete desorption of secoiridoids retained on the solids, and the filtrate finally analyzed by HPLC/DAD. The adsorption capacity of each sorbent was compared with the untreated blank sample.

### Statistical Analysis

For statistical analyses, differences between the means were analyzed for significance using the Student’s *t* test.

## Supporting Information Available

HPLC and TLC analyses, HPLC method validation, and parameters of the HPLC method used to quantify bitter secoiridoids (PDF)

## References

[ref1] Jiang M.; Cui B. W.; Wu Y. L.; Nan J. X.; Lian L. H. J. Ethnopharmacol. 2021, 264, 113391 10.1016/j.jep.2020.113391. 32931880

[ref2] Arino A.; Arberas I.; Leiton M. J.; De Renobales M.; Dominguez J. B. Z. Lebensm. Unters. Forsch. 1997, 205, 295–299. 10.1007/s002170050168.

[ref3] Fiorito S.; Epifano F.; Marchetti L.; Palumbo L.; Mascioli F.; Bastianini M.; Cardellini F.; Spogli R.; Genovese S. Food Chem. 2021, 364, 130383 10.1016/j.foodchem.2021.130383. 34153596

[ref4] Kušar A.; Šircelj H.; Bariĉeviĉ D. Isr. J. Plant Sci. 2010, 58, 291–296. 10.1560/IJPS.58.2.291.

[ref5] Takino Y.; Koshioka M.; Kawaguchi M.; Miyahara T.; Tanizawa H.; Ishi T.; Higashino M.; Hayashi T. Planta Med. 1980, 38, 351–355. 10.1055/s-2008-1074888.

[ref6] Kumar V.; Sood H.; Chauhan R. S. Nat. Prod. Res. 2015, 29, 1449–1455. 10.1080/14786419.2015.1004175. 25622657

[ref7] Lonkar S. P.; Kutlu B.; Leuteritz A.; Heinrich G. Appl. Clay Sci. 2013, 71, 8–14. 10.1016/j.clay.2012.10.009.

[ref8] Tabana L.; Tichapondwa S.; Labuschagne F.; Chirwa E. Sustainability 2020, 12, 4273 10.3390/su12104273.

[ref9] Xu H.; Xu D. C.; Wang Y. ACS Omega 2017, 2, 7185–7193. 10.1021/acsomega.7b01039. 31457297 PMC6645321

[ref10] Epifano F.; Genovese S.; Marchetti L.; Palumbo L.; Bastianini M.; Cardellini F.; Spogli R.; Fiorito S. J. Pharm. Biomed. Anal. 2020, 190, 113515 10.1016/j.jpba.2020.113515. 32798919

[ref11] Genovese S.; Epifano F.; Marchetti L.; Bastianini M.; Cardellini F.; Spogli R.; Fiorito S. J. Pharm. Biomed. Anal. 2021, 196, 113945 10.1016/j.jpba.2021.113945. 33578265

[ref12] Fiorito S.; Epifano F.; Preziuso F.; Marchetti L.; Bastianini M.; Cardellini F.; Spogli R.; Genovese S. Food Anal. Meth. 2021, 14, 1133–1139. 10.1007/s12161-020-01931-z.

[ref13] Genovese S.; Epifano F.; Marchetti L.; Bastianini M.; Cardellini F.; Spogli R.; Fiorito S. J. Food Compos. Anal. 2021, 102, 104052 10.1016/j.jfca.2021.104052. 33578265

[ref14] Fiorito S.; Epifano F.; Palumbo L.; Genovese S. Plant Foods Hum. Nutr. 2021, 76, 397–398. 10.1007/s11130-021-00911-w. 34255225

[ref15] Fiorito S.; Palumbo L.; Epifano F.; Collevecchio C.; Cardellini F.; Bastianini M.; Spogli R.; Genovese S. Food Chem. 2022, 377, 132040 10.1016/j.foodchem.2022.132040. 34999454

[ref16] Genovese S.; Epifano F.; Palumbo L.; Collevecchio C.; Bastianini M.; Cardellini F.; Spogli R.; Fiorito S. Food Chem. 2022, 387, 132901 10.1016/j.foodchem.2022.132901. 35413553

