# Supplementary material for: Leaves of Yellow Gentian (Gentiana lutea) as an Alternative Source of Bitter Secoiridoid Glycosides
Source: J Nat Prod. 2022 Aug 24;85(9):2232–5. doi: 10.1021/acs.jnatprod.2c00529 (PMC9937557; doi:10.1021/acs.jnatprod.2c00529)
Supplement: Supplementary file 1 — np2c00529_si_001.pdf [file np2c00529_si_001.pdf]

Leaves of Yellow Gentian (*Gentiana lutea*) as an Alternative Source of Bitter Secoiridoid Glycosides

Serena Fiorito[#], Francesco Epifano[#], Lucia Palumbo[#], Chiara Collevecchio[#], Fabrizio Mascioli[§], Roberto Spogli[$], Salvatore Genovese[#]*

[#]Dipartimento di Farmacia, Università "Gabriele d'Annunzio" of Chieti-Pescara, Via dei Vestini 31, 66100 Chieti Scalo (CH), Italy

[§]Enrico Toro Distilleria Srl, Via Tiburtina Valeria – Km.142,440, 65028 Tocco da Casauria (PE), Italy

[$]Prolabin & Tefarm Srl, Via dell'Acciaio 9, 06134 Perugia, Italy

Supporting information

HPLC, TLC analyses, and HPLC method validation

An Agilent 1100 (Santa Clara, CA, USA) series instrument equipped with an autosampler, a binary solvent pump, a DAD, and a Kromasil RP C18 (4.6 mm ∅ x 150 mm, 5 µm particle size) was used to accomplish HPLC anlyases. The mobile phase consisted of a double distilled H_2O - HCOOH (99.6 - 0.4%) (solvent A) and CH_3CN - HCOOH (99.6 - 0.4%) (solvent B) mixture adopting a gradient mode (flow rate 1.0 mL/min.) as the following: 0.0 - 3.0 min, from 2 % to 30 % B, 3.01 - 9.0 min. 30% B, 9.01 - 12.0 min. from 30 % to 2 % B, 12.01 – 15.0 min. 2 % B. Column temperature was fixed at 25 °C and injection volume was 20 µL. Detection wavelength for both gentiopicroside and amarogentin was 254 nm. Each solution was filtered through a 0.22 µm pore size Durapore® membrane (Merck Sigma-Aldrich, Milan, Italy) before injection into the HPLC

apparatus. Open Labs software (Agilent Technologies, Santa Clara, CA, USA) was employed for statistical analysis and data management. The HPLC method was validated following the provision given by the ICH guidelines for precision, accuracy, linearity, limits of detection (LOD), and limits of quantification (LOQ) (Table 1). The intra-day precision was determined by the injection of the standard mixture solution five times a day. For the inter-day precision, measurements were recorded once a day on three consecutive days. All these were expressed as relative standard deviations (RSDs). Precision was calculated at three concentration levels for quality control (QC) samples, namely $QC_{Low\,=}$ 1.0 µg/mL, QC_{Medium}=25.0 µg/mL, and QC_{High} = 100.0 µg/mL. Accuracy was determined by spiking samples deriving from yellow gentian leaves extracts treated with the hydrotalcite magnesium aluminium azelate (entry E) with three concentrations of the two standard compounds (low, medium, and high spikes). Calibration curves were drawn by injecting gentiopicroside and amarogentin pure standard stock solutions at the following 9 concentrations values (expressed as µg/mL): 0.5, 1.0, 5.0, 10.0, 25.0, 50.0, 75.0, 100.0 and 200.0. LODs and LOQs were obtained by injecting serial dilutions of the corresponding standard solutions, having a signal-to-noise (S/N) ratio of 3.3 and 10 as the reference, respectively. The amounts of gentiopicroside and amarogentin absorbed have been obtained by analyses of the filtrate solutions after desorption with absolute EtOH. TLC assays were accomplished using SiO_2 gel 60 F_{254} pre-coated aluminium plates (Merck Millipore, Burlington, MA; USA). Elution mixtuire consisted of a mixture of CH_2Cl_2 / MeOH /H_2O 65 / 25 /10 coupled to UV light (254 nm),$I_{2,}$ $KMnO_4$, H_2SO_4, and phosphomolibdic acid spraying detection.

Table 1. HPLC method validation more relevant parameters.

	**Compounds**	
	**1**	**2**
**Slope**	114112	112397
**Intercept**	1650	1501
**r^2**	0.9997	0.9999
**LOD (µg/mL)**	0.15	0.05
**LOQ (µg/mL)**	0.30	0.10
**Precision**		
Intra-day (n = 6)	2.1 - 3.9	2.0 – 3.8
Inter-day (n = 6)	2.3 - 4.0	2.4 – 4.1
**Accuracy**		
Intra-day (n = 6)	2.1 - 3.9	2.0 - 3.7
Inter-day (n = 6)	1.2 - 2.1	1.0 - 1.9

LOD = limit of detection; LOQ = limit of quantification
